# Wendan decoction in the treatment of nonalcoholic fatty liver disease: A systematic review and meta-analysis

**DOI:** 10.3389/fphar.2022.1039611

**Published:** 2022-10-17

**Authors:** Yufeng Zhang, Ting Liu, Lianyue Zhang, Zhongping Pu, Zheng Yan, Haibing Hua

**Affiliations:** ^1^ Department of Pulmonary and Critical Care Medicine, Jiangyin Hospital of Traditional Chinese Medicine, Jiangyin Hospital Affiliated to Nanjing University of Chinese Medicine, Jiangyin, China; ^2^ Department of Gastroenterology, Jiangyin Hospital of Traditional Chinese Medicine, Jiangyin Hospital Affiliated to Nanjing University of Chinese Medicine, Jiangyin, China; ^3^ Department of Hepatology, Jiangyin Hospital of Traditional Chinese Medicine, Jiangyin Hospital Affiliated to Nanjing University of Chinese Medicine, Jiangyin, China; ^4^ Department of Internal Medicine, Jiangyin Hospital of Traditional Chinese Medicine, Jiangyin Hospital Affiliated to Nanjing University of Chinese Medicine, Jiangyin, China

**Keywords:** wendan decoction, nonalcoholic fatty liver disease, systematic review, meta-analysis, nonalcoholic steatohepatitis

## Abstract

**Introduction:** The use of Wendan decoction (WDD) as a therapy for nonalcoholic fatty liver disease (NAFLD) has been studied in many clinical trials, and some of them showed that WDD is effective for treating this condition. However, no comprehensive research to evaluate the clinical efficacy of WDD in NAFLD patients had been performed. This systematic review and meta-analysis sought to provide an in-depth inquiry into the data currently available about the safety and effectiveness of WDD to treat NAFLD.

**Methods:** We examined the primary database for any reports of randomized controlled trials (RCTs) including WDD and its effectiveness in treating NAFLD. We used the Jadad rating scale to determine the overall quality of the selected RCTs, and we searched the Cochrane Reviewer’s Handbook for criteria for potential bias. The primary findings from the included RCTs were recorded, and the meta-analysis was performed using RevMan5.4 software developed by the Cochrane Collaboration.

**Results:** We retrieved ten RCTs that were suitable for this evaluation and included them in a systematic review and meta-analysis. The quality and risk of bias in the included RCTs were assessed. The meta-analysis showed that the total clinical effective rate was substantially greater in the WDD cohort compared with that in the control cohort, and liver function, blood lipid indices, and blood glucose-related indicators were substantially improved in the WDD-treated cohort compared with those in the control cohort. There was no significant difference in the incidence of adverse events between the two cohorts.

**Conclusion:** WDD is safe and effective for treating NAFLD, which is advantageous for the patients’ liver function as well as their blood lipid indices and blood glucose-related indicators.

## 1 Introduction

Nonalcoholic fatty liver disease (NAFLD) is an important cause of chronic liver disease in Western nations, including the United States (Mazhar, 2019). The incidence of NAFLD has increased in China due to improvements in the living standards, lifestyle changes, an aging population, and an increase in obesity, and thus, NAFLD is a prominent chronic noninfectious disease in this country (Shi et al., 2020). Current estimates suggest that NAFLD and its progressive subtype nonalcoholic steatohepatitis (NASH) affect 30% and 5%, respectively, of the current population in the United States. The most common causes of death in NASH patients are cardiovascular disease and malignancy, and it is the most rapidly increasing indication for liver transplantation (Rinella, 2015).

The most common complications linked to NAFLD include metabolic comorbidities such as dyslipidemia, diabetes mellitus (DM), and central obesity (Aller et al., 2018). Traditionally, NAFLD has a broad spectrum of subtypes that encompass nonalcoholic fatty liver (NAFL), NASH, hepatic sclerosis, hepatic fibrosis, and hepatocellular carcinoma (Diehl and Day, 2017). Using a Markov model for prediction, an estimated 33.5% of adults will have NAFLD, 27% of patients with NAFLD will have NASH, and 29% of patients with NASH will have advanced fibrosis by 2030. Patients with NAFLD currently have a significant impact on health care utilization, with an annual economic burden of $292 billion in the United States alone (Cheung et al., 2019). Early detection, aggressive management, and innovative therapies are needed because of the significant burden that is caused by this illness and the increasing consumption of health care services that result from NAFLD-related complications.

The involvement of traditional Chinese medicine (TCM) in treating NAFLD has been increasing in importance. Clinical studies and meta-analyses have indicated that TCM offers only moderate value for treating NAFLD (Shi et al., 2012; Dai L. et al., 2021). Many experiments have shown that TCM prescriptions have some beneficial effects on NAFLD in animal models (Cheng et al., 2017; Ying et al., 2021).

Wendan decoction (WDD), a TCM formulation that was first described in “Ji Yan Fang” during the Southern and Northern Dynasties, was adopted by the National Administration of TCM in 2018 and included in the “Ancient Classical Chinese Medicine Formula Catalogue (First Edition)” (Wang et al., 2021). WDD primarily consists of Ban Xia, Zhu Ru, Zhi Shi, Chen Pi, Fu Ling, and Gan Cao with the Latin names *Rhizoma Pinelliae* (RP), *Caulis Bambusae in Taeniam* (CBT), *Fructus Aurantii Immaturus* (FAI), *Pericarpium Citri Reticulatae* (PCR), *Poria* (PA), and *Radix Glycyrrhizae* (RG), respectively (Hou et al., 2021).

TCM theory indicates that the main pathogenesis of NAFLD is a weakness in the stomach and spleen or excessive nutrient intake, and the spleen and stomach cannot function normally. In TCM theory, “phlegm” is the most basic and important pathological factor of the disease (Xu et al., 2022). The main pharmacological effects of WDD are regulating qi, reducing phlegm, and benefiting the stomach, which is consistent with the TCM treatment for NAFLD.

There are currently several clinical investigations on using WDD to treat NAFLD, and some of these studies have achieved satisfactory therapeutic outcomes (Pu and Hua, 2009; Fu, 2017; Mo et al., 2018). TCM has a long history and shows an obvious effect in the treatment of related diseases. However, no single article provides a comprehensive review of WDD therapeutic effectiveness in treating NAFLD. This systematic review and meta-analysis aimed to conduct an in-depth examination of the currently available effectiveness and safety data for WDD that is used to treat patients with NAFLD.

## 2 Materials and methods

### 2.1 Ethics consideration

This systematic review included data from open databases. All the eligible studies were approved by the local institutional ethics committee. This systematic review did not directly involve patients’ private information, so additional ethics approval was not required. This review was conducted on the basis of the Preferred Reporting Items for Systematic Reviews and Meta-Analyses (PRISMA) 2020 statement (Page et al., 2021). The PRISMA 2020 checklist is shown in Supplementary File S1.

### 2.2 Datasets and research technique

We started searching the primary English and Chinese databases from database inception to 31 May 2022. The following databases were included in this research: Wanfang Data; the Chongqing VIP database; the Chinese Biomedical Literature database (SinoMed); the Chinese National Knowledge Infrastructure; the Cochrane Central Register of Controlled Trials; the Science Citation Index; EMBASE; and PUBMED.

The search term “nonalcoholic fatty liver disease,” “nonalcoholic fatty liver,” or “nonalcoholic steatohepatitis” was companied by one of the following keywords: “Wendan decoction” or “Wendan Tang.” We also screened for these keywords in the abstracts and titles of the articles. When the relevant data were missing from the abstracts, the full text was read to determine if the data were present in the article. We then manually searched the references and citations of the identified articles to determine if any other possibly relevant articles might be included. This process was repeated until no more studies were found.

### 2.3 Inclusion and exclusion criteria

The study eligibility criteria were in accordance with the participant, intervention, comparison, outcome, and study design (PICOS) principles. The inclusion criteria were as follows: study subjects met the criteria for NAFLD diagnosis, which might also apply to NAFL or NASH (P); WDD was used in the experimental group, and WDD prescription included at least RP, CBT, FAI, PCR, and PA (I); the treatment modality of choice for the control cohort was conventional Western medicine treatment (CWMT), in the absence of TCM (C); all the outcome indicators from the included studies were retrieved and evaluated, a meta-analysis was performed when the indicators could be subjected to a meta-analysis, and a descriptive analysis was performed when a meta-analysis could not be performed (O); and the research was conducted using a randomized controlled trial (RCT) approach (S).

The exclusion criteria were as follows: 1) for duplicate publications, only the most recent edition or the publication that had the most comprehensive data was included and the others were disregarded; 2) animal experiments; 3) abstracts, reviews, and case reports; and 4) articles in which there were insufficient data collected to properly measure the effect.

### 2.4 Data retrieval and quality evaluation

The data were compiled and analyzed independently by two reviewers. The data collected included the first author, the publication year, the total number of patients assigned to each cohort, the primary component of WDD prescriptions, the methodologies of intervention administered to the control and experimental groups, the outcomes, and information for quality assessment. Disagreements about the research details were resolved through discussion until the issue was settled by consensus.

The Jadad rating scale and the Cochrane Reviewer’s Handbook were used when evaluating the quality of the chosen RCTs and to assess the potential for bias in these investigations (Jadad et al., 1996; Higgins et al., 2011). To calculate the Jadad scores, each RCT was evaluated using the following three prerequisites: randomization (0–2 points), blinding (0–2 points), and dropouts and withdrawals (0–1 points). If any of these terms was referenced in the research, it was awarded one point. One point was added to the total score if details were included on the technique used to produce the randomization sequence or on the blinding procedure and if this technique and procedure were appropriate, but one point was subtracted if the method was inappropriate. On the quality scale, 0 to 5 points were awarded, and higher scores reflected more accurate reporting. Accuracy scores were classified into the following two categories: low quality, which received fewer than 3 points; and excellent quality, which received 3 points or more (Xia et al., 2020; Zhang et al., 2020). The Cochrane classification, which includes seven criteria, was used to evaluate the potential for bias. These criteria included incomplete outcome measures, preferential outcome reporting, allocation concealment, random number generation, patient blinding, assessor blinding, and other potential biases (Wang et al., 2020).

### 2.5 Statistical analyses

Both the systematic review and the meta-analysis were performed using the RevMan5.4 software by the Cochrane Collaboration [Review Manager (RevMan) (Computer program). Version 5.4. The Cochrane Collaboration, 2020]. Dichotomous data are presented as the odds ratios (OR) with a 95% confidence interval (CI). Continuous data are presented as the mean difference (MD) with the 95% CI.

The Q-test was used to determine whether the data showed heterogeneity (*p* value and *I* ^*2*^); this test describes the percentage of variability in the effect and estimates the contribution of heterogeneity. The presence of heterogeneity among the studies was shown by a significant Q-statistic (*p* less than 0.10). Studies were deemed to have no heterogeneity if their I^2^ was <50%, whereas those with an I^2^ ≥ 50% were considered to be heterogeneous. The fixed-effects model was chosen for the pooling technique if there was no evidence of considerable heterogeneity, and the random-effects model was used as a suitable alternative for all other cases (Higgins and Thompson, 2002; Liu et al., 2022).

When heterogeneity was high and the number of included studies was sufficient, we assessed the interventions (monotherapy or combined CWMT), different controls (placebo, CWMT, or no interventions), and various treatment durations to perform a subgroup analysis (Low et al., 2019). Sensitivity analysis was performed on the basis of the methodological quality (low-quality studies were omitted and the results of this meta-analysis were re-evaluated), statistical model (the random-effects or fixed-effects model was used for the analysis), and sample size (studies with a smaller sample size were omitted and the outcomes of this meta-analysis were re-evaluated), (Taeuber et al., 2021). We used a funnel plot to analyze the data and assess whether there was any publication bias when more relevant articles were considered in the meta-analysis.

All probabilities (*p*-values) that were presented were two-sided, and a *p*-value <0.05 was considered to be statistically significant.

## 3 Results

### 3.1 Research selection

**FIGURE 1:**
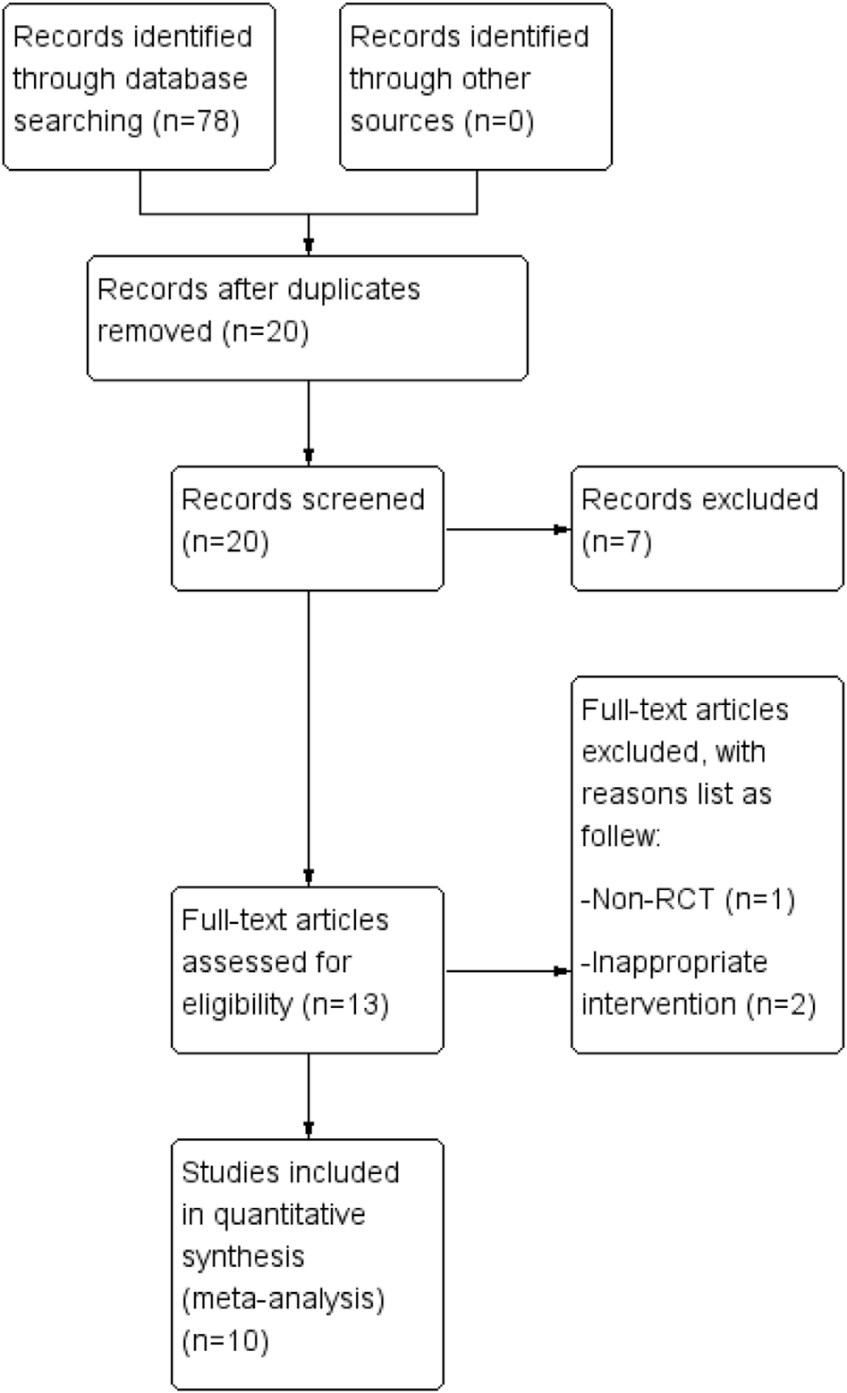
The procedure for study selection. A database search yielded 78 articles. The systematic review includes ten randomized controlled trials.

After searching the databases, 78 studies were obtained, and only 20 studies remained after duplicates were removed. After reviewing each study’s title and abstract, we excluded seven unrelated studies. When the complete text was reviewed, three additional studies (Pu et al., 2009; Chen, 2018; Sun et al., 2020) were not included for several reasons, as follows: one study (Pu et al., 2009) had no control group; and two studies (Chen, 2018; Sun et al., 2020) did not include FAI in the TCM prescription. Thus, ten RCTs (Pu and Hua, 2009; Hu et al., 2012; Huang et al., 2012; Pan, 2013; Fu, 2017; Chen et al., 2018; Hui, 2018; Mo et al., 2018; Wang et al., 2018; Cai, 2020) were included in the systematic review. Figure 1 shows the procedure that was used to identify the relevant literature and outcomes.

### 3.2 Overview of the included studies

Ten eligible RCTs (Pu and Hua, 2009; Hu et al., 2012; Huang et al., 2012; Pan, 2013; Fu, 2017; Chen et al., 2018; Hui, 2018; Mo et al., 2018; Wang et al., 2018; Cai, 2020) were identified. There were 875 participants included in the ten RCTs, and all of these studies were performed in China. The ten RCTs were all single-center studies. Patients in two RCTs (Wang et al., 2018; Cai, 2020) had NAFLD combined with DM. The experimental group treatments included TCM prescriptions. One RCT (Fu, 2017) used WDD including only RP, CBT, FAI, PCR, PA, and RG, and other RCTs (Pu and Hua, 2009; Hu et al., 2012; Huang et al., 2012; Pan, 2013; Chen et al., 2018; Hui, 2018; Mo et al., 2018; Wang et al., 2018; Cai, 2020) used WDD including at least RP, CBT, FAI, PCR and PA. The experimental group treatment in three studies (Hui, 2018; Wang et al., 2018; Cai, 2020) included CWMT. The control group treatment included CWMT, and polyene phosphatidylcholine capsules were used in four studies (Pu and Hua, 2009; Hu et al., 2012; Pan, 2013; Hui, 2018). Additionally, one study (Huang et al., 2012) used simvastatin tablets and trivitamin B tablets; one study (Fu, 2017) used xuezhikang capsules; one RCT (Mo et al., 2018) used silibinin capsules; one study (Chen et al., 2018) used atorvastatin calcium tablets; one RCT (Wang et al., 2018) used saxagliptin tablets; and one study (Cai, 2020) used acarbose tablets. One RCT (Hu et al., 2012) showed two time points (3 months and 6 months) for the indicators, and we selected the 6-month indicators for these meta-analyses. Table 1 provides an outline of the primary characteristics of the included research. Table 2 shows an overview of the TCM prescriptions that were administered in the experimental groups in each study. Table 3 presents an overview of the quality control that was applied to TCM prescriptions.

**TABLE 1:** Summary of RCTs that investigated WDD for NAFLD.

Study year [ref]	Country	Sample size (Experimental/Control)	Mean age (years) (Experimental/Control)	Experimental	Control	Duration
Pu et al., 2009, (Pu and Hua, 2009)	China	100 (53/47)	18–65(36.9)/20–66(37.1)	Modified WDD	Polyene Phosphatidylcholine Capsules	2 months
Huang et al., 2012, (Huang et al., 2012)	China	60 (30/30)	44 ± 3.4/46 ± 3.5	Modified Huanglian WDD	Simvastatin Tablets + Trivitamins B Tablets	15 days
Hu et al., 2012, (Hu et al., 2012)	China	100 (50/50)	43 ± 15.6/42 ± 14.8	Chaihu WDD	Polyene Phosphatidylcholine Capsules	3 months/6 months
Pan 2013, (Pan, 2013)	China	61 (36/25)	20–68(38.6)/22–65(35.9)	Modified WDD	Polyene Phosphatidylcholine Capsules	2 months
Fu 2017, (Fu, 2017)	China	60 (30/30)	30–60	WDD	Xuezhikang Capsules	3 months
Mo et al., 2018, (Mo et al., 2018)	China	120 (60/60)	n.r	Huanglian WDD	Silibinin Capsules	2 months
Hui, 2018, (Hui, 2018)	China	114 (57/57)	44.38 ± 5.14/42.63 ± 6.49	Polyene Phosphatidylcholine Capsules + Modified WDD	Polyene Phosphatidylcholine Capsules	3 months
Chen et al., 2018, (Chen et al., 2018)	China	100 (50/50)	33.71 ± 6.37/34.07 ± 6.86	Huanglian WDD	Atorvastatin Calcium Tablets	2 months
Wang et al., 2018, (Wang et al., 2018)	China	96 (48/48)	54.21 ± 3.71/54.27 ± 3.45	Saxagliptin Tablets + Modified WDD	Saxagliptin Tablets	3 months
Cai, 2020, (Cai, 2020)	China	64 (32/32)	55.16 ± 6.39/56.37 ± 6.41	Acarbose Tablets + Modified WDD	Acarbose Tablets	3 months

RCT, randomized controlled trial; WDD, wendan decoction; NAFLD, nonalcoholic fatty liver disease; n. r, not reported.

**TABLE 2:** Composition of the TCM prescriptions.

Study year [ref]	TCM prescriptions	Composition of TCM prescriptions		
		Latin name	English name	Chinese name
Pu et al., 2009, (Pu and Hua, 2009)	Modified WDD	**Rhizoma Pinelliae (RP), Caulis Bambusae in Taeniam (CBT), Fructus Aurantii Immaturus (FAI), Pericarpium Citri Reticulatae (PCR), Poria (PA), Radix Glycyrrhizae (RG),** Rhizoma Alismatis, Fructus Crataegi, Pericarpium Arecae, Massa Medicata Fermentata, Radix Salviae Miltiorrhizae, Rhizoma Zingiberis Recens, Fructus Jujube	**Pinellia Rhizome, Bamboo Shavings, Immature Bitter Orange, Tangerine Peel, Tuckahoe, Licorice Root,** Alisma Rhizome, Crataegus Fruit, Areca HuskMassa, Fermentata, Red Sage Root, Fresh Ginger Rhizome, Jujube	**Ban Xia, Zhu Ru, Zhi Shi, Chen Pi, Fu Ling, Gan Cao,** Ze Xie, Shan Zha, Da Fu Pi, Shen Qu, Dan Shen, Sheng Jiang, Da Zao
Huang et al., 2012, (Huang et al., 2012)	Modified Huanglian WDD	**Rhizoma Pinelliae (RP), Caulis Bambusae in Taeniam (CBT), Fructus Aurantii Immaturus (FAI), Pericarpium Citri Reticulatae (PCR), Poria (PA), Radix Glycyrrhizae (RG),** Arisaemae cum Bile, Rhizoma Coptidis, Fructus Amomi, Cortex Cinnamomi, Fructus Schisandrae, Pericarpium Trichosanthis, Herba Artemisiae Scopariae, Fructus Crataegi, Rhizoma Polygonati, Rhizoma Curcumae Longae, Radix Polygoni Multiflori	**Pinellia Rhizome, Bamboo Shavings, Immature Bitter Orange, Tangerine Peel, Tuckahoe, Licorice Root,** Pulvis Arisaema, Coptis Rhizome, Amomum Fruit, Cinnamon Bark, Schisandra Fruit, Trichosanthis Peel, Virgate Wormwood, Crataegus Fruit, Polygonatum Rhizome, Turmeric Rhizome, Fleeceflower Root	**Ban Xia, Zhu Ru, Zhi Shi, Chen Pi, Fu Ling, Gan Cao,** Dan Nan Xing, Huang Lian, Sha Ren, Rou Gui, Wu Wei Zi, Gua Lou Pi, Yin Chen, Shan Zha, Huang Jing, Jiang Huang, He Shou Wu
Hu et al., 2012, (Hu et al., 2012)	Chaihu WDD	**Rhizoma Pinelliae (RP), Caulis Bambusae in Taeniam (CBT), Fructus Aurantii Immaturus (FAI), Pericarpium Citri Reticulatae (PCR), Poria (PA),** Radix Bupleuri, Radix Scutellariae, Rhizoma Alismatis, Rhizoma Polygoni Cuspidati, Rhizoma Zingiberis Recens, Fructus Jujube	**Pinellia Rhizome, Bamboo Shavings, Immature Bitter Orange, Tangerine Peel, Tuckahoe,** Bupleurum Root, Scute, Alisma Rhizome, Bushy Knotweed Rhizome, Fresh Ginger Rhizome, Jujube	**Ban Xia, Zhu Ru, Zhi Shi, Chen Pi, Fu Ling,** Chai Hu, Huang Qin, Ze Xie, Hu Zhang, Sheng Jiang, Da Zao
Pan, 2013, (Pan, 2013)	Modified WDD	**Rhizoma Pinelliae (RP), Caulis Bambusae in Taeniam (CBT), Fructus Aurantii Immaturus (FAI), Pericarpium Citri Reticulatae (PCR), Poria (PA), Radix Glycyrrhizae (RG),** Rhizoma Alismatis, Fructus Crataegi, Radix Salviae Miltiorrhizae, Rhizoma Zingiberis Recens, Fructus Jujube	**Pinellia Rhizome, Bamboo Shavings, Immature Bitter Orange, Tangerine Peel, Tuckahoe, Licorice Root,** Alisma Rhizome, Crataegus Fruit, Red Sage Root, Fresh Ginger Rhizome, Jujube	**Ban Xia, Zhu Ru, Zhi Shi, Chen Pi, Fu Ling, Gan Cao,** Ze Xie, Shan Zha, Dan Shen, Sheng Jiang, Da Zao
Fu, 2017, (Fu, 2017)	WDD	**Rhizoma Pinelliae (RP), Caulis Bambusae in Taeniam (CBT), Fructus Aurantii Immaturus (FAI), Pericarpium Citri Reticulatae (PCR), Poria (PA), Radix Glycyrrhizae (RG)**	**Pinellia Rhizome, Bamboo Shavings, Immature Bitter Orange, Tangerine Peel, Tuckahoe, Licorice Root**	**Ban Xia, Zhu Ru, Zhi Shi, Chen Pi, Fu Ling, Gan Cao**
Mo et al., 2018, (Mo et al., 2018)	Huanglian WDD	**Rhizoma Pinelliae (RP), Caulis Bambusae in Taeniam (CBT), Fructus Aurantii Immaturus (FAI), Pericarpium Citri Reticulatae (PCR), Poria (PA), Radix Glycyrrhizae (RG),** Rhizoma Coptidis, Rhizoma Zingiberis Recens	**Pinellia Rhizome, Bamboo Shavings, Immature Bitter Orange, Tangerine Peel, Tuckahoe, Licorice Root,** Coptis Rhizome, Fresh Ginger Rhizome	**Ban Xia, Zhu Ru, Zhi Shi, Chen Pi, Fu Ling, Gan Cao,** Huang Lian, Sheng Jiang
Hui, 2018, (Hui, 2018)	Modified WDD	**Rhizoma Pinelliae (RP), Caulis Bambusae in Taeniam (CBT), Fructus Aurantii Immaturus (FAI), Pericarpium Citri Reticulatae (PCR), Poria (PA), Radix Glycyrrhizae (RG)**, Fructus Crataegi, Semen Cassiae	**Pinellia Rhizome, Bamboo Shavings, Immature Bitter Orange, Tangerine Peel, Tuckahoe Licorice Root,** Crataegus Fruit, Cassia Seeds	**Ban Xia, Zhu Ru, Zhi Shi, Chen Pi, Fu Ling, Gan Cao,** Shan Zha, Jue Ming Zi
Chen, 2018, (Chen et al., 2018)	Huanglian WDD	**Rhizoma Pinelliae (RP), Caulis Bambusae in Taeniam (CBT), Fructus Aurantii Immaturus (FAI), Pericarpium Citri Reticulatae (PCR), Poria (PA), Radix Glycyrrhizae (RG)**, Rhizoma Coptidis, Fructus Jujube, Radix Curcumae, Rhizoma Zingiberis Recens, Radix Aucklandiae, Radix Salviae Miltiorrhizae, Radix Notoginseng, Radix Angelicae Dahuricae	**Pinellia Rhizome, Bamboo Shavings, Immature Bitter Orange, Tangerine Peel, Tuckahoe, Licorice Root,** Coptis Rhizome Jujube, Curcuma Tuber, Fresh Ginger Rhizome, Aucklandia, Red Sage Root, Notoginseng Root, Angelica Root	**Ban Xia, Zhu Ru, Zhi Shi, Chen Pi, Fu Ling, Gan Cao,** Huang Lian, Da Zao, Yu Jin, Sheng Jiang, Mu Xiang, Dan Shen, San Qi, Bai Zhi
Wang et al., 2018, (Wang et al., 2018)	Modified WDD	**Rhizoma Pinelliae (RP), Caulis Bambusae in Taeniam (CBT), Fructus Aurantii Immaturus (FAI), Pericarpium Citri Reticulatae (PCR), Poria (PA), Radix Glycyrrhizae (RG),** Fructus Crataegi, Radix Salviae Miltiorrhizae, Rhizoma Alismatis, Rhizoma Zingiberis Recens, Fructus Jujube	**Pinellia Rhizome, Bamboo Shavings, Immature Bitter Orange, Tangerine Peel, Tuckahoe, Licorice Root,** Crataegus Fruit, Red Sage Root, Alisma Rhizome, Fresh Ginger Rhizome, Jujube	**Ban Xia, Zhu Ru, Zhi Shi, Chen Pi, Fu Ling, Gan Cao**, Shan Zha, Dan Shen, Ze Xie, Sheng Jiang, Da Zao
Cai, 2020, (Cai, 2020)	Modified WDD	**Rhizoma Pinelliae (RP), Caulis Bambusae in Taeniam (CBT), Fructus Aurantii Immaturus (FAI), Pericarpium Citri Reticulatae (PCR), Poria (PA), Radix Glycyrrhizae (RG),** Fructus Crataegi, Radix Salviae Miltiorrhizae, Rhizoma Alismatis, Rhizoma Zingiberis Recens, Fructus Jujube	**Pinellia Rhizome, Bamboo Shavings, Immature Bitter Orange, Tangerine Peel, Tuckahoe, Licorice Root,** Crataegus Fruit, Red Sage Root, Alisma Rhizome, Fresh Ginger Rhizome, Jujube	**Ban Xia, Zhu Ru, Zhi Shi, Chen Pi, Fu Ling, Gan Cao,** Shan Zha, Dan Shen, Ze Xie, Sheng Jiang, Da Zao

TCM, traditional Chinese medicine; WDD, wendan decoction.

**TABLE 3:** Quality control of the TCM prescriptions.

Study year [ref]	TCM prescriptions	Source	Major ingredient dose
Pu et al., 2009, (Pu and Hua, 2009)	Modified WDD	Jiangyin Hospital of Traditional Chinese Medicine, Jiangsu, China	Rhizoma Pinelliae (RP) 20 g, Caulis Bambusae in Taeniam (CBT) 15 g, Fructus Aurantii Immaturus (FAI) 12 g, Pericarpium Citri Reticulatae (PCR) 15 g, Poria (PA) 10 g, Radix Glycyrrhizae (RG) 6 g
Huang et al., 2012, (Huang et al., 2012)	Modified Huanglian WDD	Guangdong Second Traditional Chinese Medicine Hospital, Guangdong, China	Rhizoma Pinelliae (RP) 15 g, Caulis Bambusae in Taeniam (CBT) 20 g, Fructus Aurantii Immaturus (FAI) 15 g, Pericarpium Citri Reticulatae (PCR) 10 g, Poria (PA) 30 g, Radix Glycyrrhizae (RG) 10 g
Hu et al., 2012, (Hu et al., 2012)	Chaihu WDD	Foshan Hospital of Traditional Chinese Medicine, Guangdong, China	Rhizoma Pinelliae (RP) 9 g, Caulis Bambusae in Taeniam (CBT) 10 g, Fructus Aurantii Immaturus (FAI) 10 g, Pericarpium Citri Reticulatae (PCR) 10 g, Poria (PA) 15 g
Pan, 2013, (Pan, 2013)	Modified WDD	Jiangning Hospital of Traditional Chinese Medicine, Jiangsu, China	Rhizoma Pinelliae (RP) 20 g, Caulis Bambusae in Taeniam (CBT) 15 g, Fructus Aurantii Immaturus (FAI) 12 g, Pericarpium Citri Reticulatae (PCR) 15 g, Poria (PA) 10 g, Radix Glycyrrhizae (RG) 6 g
Fu, 2017, (Fu, 2017)	WDD	Jilin Provincial Hospital of Traditional Chinese Medicine, Jilin, China	Rhizoma Pinelliae (RP) 10 g, Caulis Bambusae in Taeniam (CBT) 10 g, Fructus Aurantii Immaturus (FAI) 10 g, Pericarpium Citri Reticulatae (PCR) 10 g, Poria (PA) 20 g, Radix Glycyrrhizae (RG) 10 g
Mo et al., 2018, (Mo et al., 2018)	Huanglian WDD	Foshan Hospital of Traditional Chinese Medicine, Guangdong, China	Rhizoma Pinelliae (RP) 9 g, Caulis Bambusae in Taeniam (CBT) 10 g, Fructus Aurantii Immaturus (FAI) 10 g, Pericarpium Citri Reticulatae (PCR) 10 g, Poria (PA) 30 g, Radix Glycyrrhizae (RG) 5 g
Hui 2018, (Hui, 2018)	Modified WDD	Yulin Hospital of Traditional Chinese Medicine, Shaanxi, China	Rhizoma Pinelliae (RP) 10 g, Caulis Bambusae in Taeniam (CBT) 10 g, Fructus Aurantii Immaturus (FAI) 10 g, Pericarpium Citri Reticulatae (PCR) 10 g, Poria (PA) 15 g, Radix Glycyrrhizae (RG) 3 g
Chen et al., 2018, (Chen et al., 2018)	Huanglian WDD	Tiandong Hospital of Traditional Chinese Medicine, Guangxi, China	Rhizoma Pinelliae (RP) 10 g, Caulis Bambusae in Taeniam (CBT) 15 g, Fructus Aurantii Immaturus (FAI) 10 g, Pericarpium Citri Reticulatae (PCR) 10 g, Poria (PA) 30 g, Radix Glycyrrhizae (RG) 3 g
Wang et al., 2018, (Wang et al., 2018)	Modified WDD	Weihai Municipal Hospital, Shandong, China	Rhizoma Pinelliae (RP) 20 g, Caulis Bambusae in Taeniam (CBT) 15 g, Fructus Aurantii Immaturus (FAI) 12 g, Pericarpium Citri Reticulatae (PCR) 15 g, Poria (PA) 10 g, Radix Glycyrrhizae (RG) 6 g
Cai 2020, (Cai, 2020)	Modified WDD	The Third People’s Hospital of Hechuan District, Chongqing, China	Rhizoma Pinelliae (RP) 20 g, Caulis Bambusae in Taeniam (CBT) 15 g, Fructus Aurantii Immaturus (FAI) 12 g, Pericarpium Citri Reticulatae (PCR) 15 g, Poria (PA) 10 g, Radix Glycyrrhizae (RG) 6 g

TCM, traditional Chinese medicine; WDD, wendan decoction.

### 3.3 Methodological quality

**FIGURE 2:**
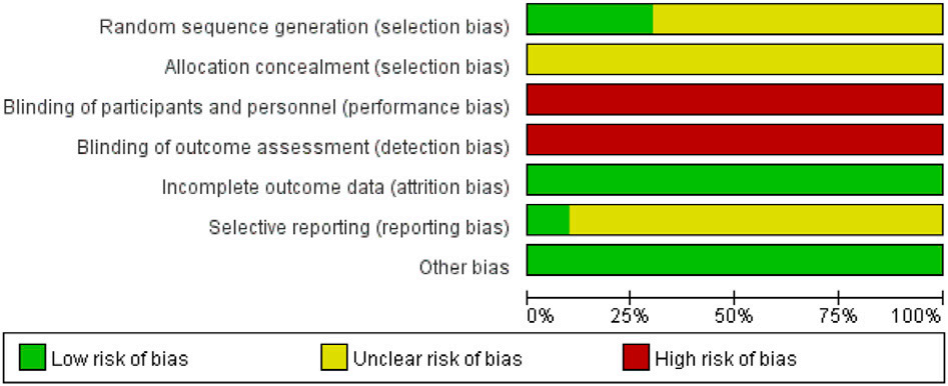
Risk of bias. The risk of bias item (percentage) across all examined studies.

Three RCTs (Huang et al., 2012; Chen et al., 2018; Mo et al., 2018) used a table of random digits *via* random sequence generation, whereas the other RCTs referred only to randomization. Ten RCTs did not introduce allocation concealment or blinding, one RCT (Fu, 2017) introduced dropouts and withdrawals, and none of the studies indicated the presence of selective reporting (Figure 2; Table 4, Supplementary Figure S1). The Jadad rating score provided points ranging from 1 to 5. The Jadad rating score was 2 points in four of the RCTs (Huang et al., 2012; Fu, 2017; Chen et al., 2018; Mo et al., 2018) and 1 point in the other six RCTs (Table 4).

**TABLE 4:** Risk of bias and quality of the included RCTs.

Study year [ref]	Random sequence generation	Allocation concealment	Blinding of patient	Blinding of assessor	Incomplete outcome data	Selective reporting	Other bias	Jadad score
Pu et al., 2009, (Pu and Hua, 2009)	U	U	H	H	L	U	L	1
Huang et al., 2012, (Huang et al., 2012)	L	U	H	H	L	U	L	2
Hu et al., 2012, (Hu et al., 2012)	U	U	H	H	L	U	L	1
Pan 2013, (Pan, 2013)	U	U	H	H	L	U	L	1
Fu 2017, (Fu, 2017)	U	U	H	H	L	L	L	2
Mo et al., 2018, (Mo et al., 2018)	L	U	H	H	L	U	L	2
Hui, 2018, (Hui, 2018)	U	U	H	H	L	U	L	1
Chen et al., 2018, (Chen et al., 2018)	L	U	H	H	L	U	L	2
Wang et al., 2018, (Wang et al., 2018)	U	U	H	H	L	U	L	1
Cai, 2020, (Cai, 2020)	U	U	H	H	L	U	L	1

RCT, randomized controlled trial; L, low risk of bias; H, high risk of bias; U, unclear (uncertain risk of bias).

### 3.4 Outcomes

Seven RCTs (Pu and Hua, 2009; Huang et al., 2012; Pan, 2013; Fu, 2017; Chen et al., 2018; Hui, 2018; Wang et al., 2018) evaluated the overall clinical effective rate.

Five studies (Pu and Hua, 2009; Huang et al., 2012; Chen et al., 2018; Hui, 2018; Wang et al., 2018) compared serum alanine aminotransferase (ALT), five studies (Pu and Hua, 2009; Huang et al., 2012; Chen et al., 2018; Hui, 2018; Wang et al., 2018) compared serum aspartate aminotransferase (AST), and two RCTs (Hu et al., 2012; Wang et al., 2018) compared serum γ-glutamyl transpeptidase (GGT) levels.

Six studies (Hu et al., 2012; Huang et al., 2012; Fu, 2017; Chen et al., 2018; Hui, 2018; Wang et al., 2018) compared serum total cholesterol (TC), six studies (Hu et al., 2012; Huang et al., 2012; Fu, 2017; Chen et al., 2018; Hui, 2018; Wang et al., 2018) compared serum triglyceride (TG), three studies (Fu, 2017; Chen et al., 2018; Hui, 2018) compared serum low-density lipoprotein cholesterol (LDL-C), and two studies (Fu, 2017; Hui, 2018) compared serum high-density lipoprotein cholesterol (HDL-C) levels.

Three studies (Hui, 2018; Wang et al., 2018; Cai, 2020) compared fasting blood glucose (FBG) levels, two studies (Hui, 2018; Wang et al., 2018) compared fasting serum insulin (FINS) levels; and two studies (Hui, 2018; Wang et al., 2018) compared the homeostatic model assessment insulin resistance (HOMA-IR).

One RCT (Hu et al., 2012) evaluated the type B ultrasonic score and clinical symptom score, one study (Mo et al., 2018) compared controlled attenuation parameter (CAP), one study (Hui, 2018) compared superoxide dismutase (SOD) and malondialdehyde (MDA), one study (Wang et al., 2018) compared glycosylated hemoglobin type A1c (HbA1c), and one study (Cai, 2020) compared 2-h postprandial blood glucose (PBG).

Four RCTs mentioned the presence of adverse complications (Fu, 2017; Chen et al., 2018; Wang et al., 2018; Cai, 2020), and the other studies did not mention whether there were adverse reactions.

Table 5 shows an overview of the key results and outcomes.

**TABLE 5:** Main outcomes of included RCTs.

Study year [ref]	Main outcomes	Main results (effect size)	Adverse events
Pu et al., 2009, (Pu and Hua, 2009)	1) Total effective rate of clinical efficacy 2) Liver function indices ALT AST	OR, 3.72 [1.38, 10.05] MD, -13.84 [-21.15, -6.53] MD, -15.00 [-24.22, -5.78]	n.r
Huang et al., 2012, (Huang et al., 2012)	1) Total effective rate of clinical efficacy 2) Liver function indices ALT AST 3) Blood lipid indices TC TG	OR, 3.86 [0.93, 16.05] MD, -12.19 [-17.31, -7.07] MD, -9.89 [-14.42, -5.36] MD, 0.01 [-0.45, 0.47] MD, -0.56 [-0.94, -0.18]	n.r
Hu et al., 2012, (Hu et al., 2012)	1) Liver function indices ALT AST GGT 2) Blood lipid indices TC TG 3) Type B ultrasonic score 4) Clinical symptom score	MD, 1.00 [-4.47, 6.47] MD, -2.00 [-7.12, 3.12] MD, 1.00 [-4.53, 6.53] MD, -1.01 [-1.76, -0.26] MD, -0.40 [-0.63, -0.17] MD, -0.10 [-0.40, 0.20] MD, -0.90 [-1.18, -0.62]	n.r
Pan, 2013, (Pan, 2013)	1) Total effective rate of clinical efficacy	OR, 4.13 [1.20, 14.25]	n.r
Fu, 2017, (Fu, 2017)	1) Total effective rate of clinical efficacy 2) Blood lipid index TC TG LDL-C HDL-C	OR, 4.57 [1.45, 14.39] MD, 0.43 [-0.02, 0.88] MD, -0.54 [-0.93, -0.15] MD, 0.40 [-0.10, 0.90] MD, -0.13 [-0.20, -0.06]	Control: Gastrointestinal discomfort (n = 2)
Mo et al., 2018, (Mo et al., 2018)	1) CAP	MD, -47.01 [-59.68, -34.34]	n.r
Hui, 2018, (Hui, 2018)	1) Total effective rate of clinical efficacy 2) Blood lipid index TC TG LDL-C HDL-C 3) Blood glucose-related indicators FPG FINS HOMA-IR 4) Oxidative stress indicators SOD MDA	OR, 9.14 [1.10, 75.71] MD, -1.29 [-1.97, -0.61] MD, -0.57 [-0.80, -0.34] MD, -0.36 [-0.78, 0.06] MD, 0.07 [-0.19, 0.33] MD, -1.16 [-1.67, -0.65] MD, -2.55 [-3.31, -1.79] MD, -0.77 [-1.04, -0.50] MD, 12.74 [6.67, 18.81] MD, -1.24 [-1.90, -0.58]	n.r
Chen et al., 2018, (Chen et al., 2018)	1) Total effective rate of clinical efficacy 2) Liver function index ALT AST 3) Blood lipid index TC TG LDL-C	OR, 2.54 [0.81, 7.94] MD, -20.42 [-26.56, -14.28] MD, -11.72 [-16.74, -6.70] MD, -1.05 [-1.22, -0.88] MD, -0.80 [-0.96, -0.64] MD, [-0.96, -0.64]	Control: Mild diarrhea (n = 1)
Wang et al., 2018, (Wang et al., 2018)	1) Total effective rate of clinical efficacy 2) Liver function index ALT AST GGT 3) Blood lipid index TC TG 4) Blood glucose-related indicators FPG FINS HOMA-IR HbA1c	OR, 6.18 [1.64, 23.22] MD, -17.56 [-24.48, -10.64] MD, -18.37 [-24.99, -11.75] MD, -20.59 [-28.62, -12.56] MD, -0.85 [-1.33, -0.37] MD, -0.41 [-0.63, -0.19] MD, -1.93 [-2.72, -1.14] MD, -2.48 [-3.30, -1.66] MD, -2.05 [-2.25, -1.85] MD, -0.85 [-1.62, -0.08]	Experimental: Gastrointestinal discomfort (n = 2) Nausea and vomiting (n = 3) Glucopenia (n = 1) Dizziness (n = 1) Control: Gastrointestinal discomfort (n = 2) Nausea and vomiting (n = 1) Dizziness (n = 2)
Cai, 2020, (Cai, 2020)	1) Blood glucose-related indicators FBG PBG	MD, -7.53 [-8.92, -6.14] MD, -9.49 [-11.49, -7.49]	Experimental: Gastrointestinal discomfort (n = 1) Control: Gastrointestinal discomfort (n = 2) Nausea (n = 2) Dizziness (n = 1)

RCT, randomized controlled trial; ALT, alanine aminotransferase; AST, aspartate aminotransferase; TC, total cholesterol; TG, triglyceride; GGT, γ-glutamyl transpeptidase; LDL-C, low-density lipoprotein cholesterol; HDL-C, high-density lipoprotein cholesterol; CAP, controlled attenuation parameter; FBG, fasting blood glucose; FINS, fasting serum insulin; HOMA-IR, homeostatic model assessment insulin resistance; SOD, superoxide dismutase; MDA, malondialdehyde; HbA1c, glycosylated hemoglobin type A1c; PBG, 2-h postprandial blood glucose; OR, odds ratio; MD, mean difference; n. r, not reported.

### 3.5 Meta-analysis

#### 3.5.1 Subgroup analysis and sensitivity analysis

We first assessed the heterogeneity on the basis of interventions, controls, and treatment duration. When the heterogeneity was low, subgroup analysis was not performed. Sensitivity analysis was performed on the basis of the methodological quality, statistical model, and sample size. The sensitivity analysis demonstrated that the robustness and reliability of the pooled results were fair.

#### 3.5.2 Total clinical effective rate

**FIGURE 3:**
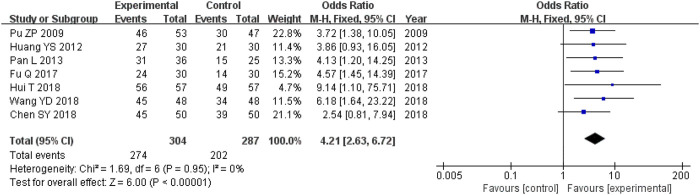
Comparative forest plot: total clinical effective rate. The pooled OR was 4.21 (95% CI 2.63–6.72, *p* < 0.00001). The total clinical effective rate was significantly higher in the experimental cohort compared with that in the control cohort. OR, odds ratio; CI, confidence interval.

There were 591 participants enrolled into the seven trials (Pu and Hua, 2009; Huang et al., 2012; Pan, 2013; Fu, 2017; Chen et al., 2018; Hui, 2018; Wang et al., 2018) that examined the total clinical effective rate (TCM syndrome). Among these participants, 304 were in the experimental cohort and 287 were in the control cohort. There was homogeneity throughout all seven trials (heterozygosity test, Χ^2^ = 1.69, *p* = 0.95, *I* ^*2*^ = 0%). After using the fixed-effects model to merge OR values, the pooled OR was 4.21 (95% CI 2.63–6.72, *p* < 0.00001). This demonstrated that the experimental cohort had a significantly greater total clinical effective rate compared with that of the control cohort (Figure 3).

#### 3.5.3 Liver function indices

**FIGURE 4:**
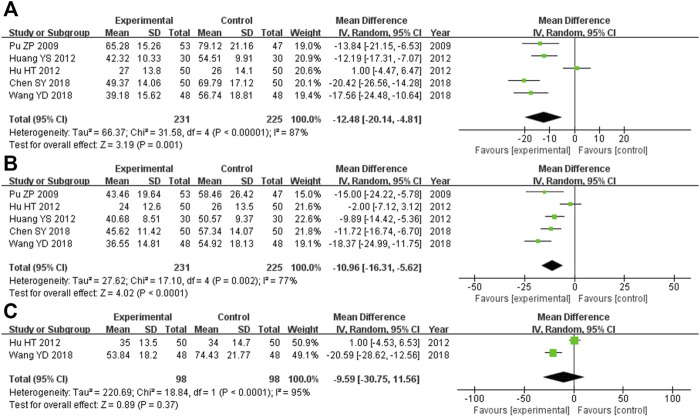
Comparative forest plot: liver function indices. **(A)** The pooled MD was −12.48 (95% CI −20.14 to −4.81, *p* = 0.001). The ALT levels in the experimental cohort were significantly lower than those in the control cohort. **(B)** The pooled MD was −10.96 (95% CI −16.31 to −5.62, *p* < 0.0001). The AST levels in the experimental cohort were significantly lower than those in the control cohort. **(C)** The pooled MD was −9.59 (95% CI −30.75 to 11.56, *p* = 0.37). There was no significant variation between the experimental and control cohorts for GGT. MD, mean difference; ALT, alanine aminotransferase; CI, confidence interval; AST, aspartate aminotransferase; GGT, γ-glutamyl transpeptidase.

There were 456 participants across the five trials (Pu and Hua, 2009; Huang et al., 2012; Chen et al., 2018; Hui, 2018; Wang et al., 2018) that compared the ALT levels, with 231 in the experimental cohort and 225 in the control cohort. Heterozygosity was observed across all five trials (heterozygosity test, Χ^2^ = 31.58, *p* < 0.00001, *I* ^*2*^ = 87%). After applying the random-effects model to combine the MD data, the pooled MD was −12.48 (95% CI −20.14 to −4.81, *p* = 0.001). This suggested that the ALT levels in the experimental cohort were significantly lower than those in the control cohort (Figure 4A).

Overall, there were 456 participants across the five RCTs (Pu and Hua, 2009; Huang et al., 2012; Chen et al., 2018; Hui, 2018; Wang et al., 2018) that compared AST levels, and 231 of them were in the experimental cohort, while the other 225 were controls. There was heterozygosity throughout all five trials (heterozygosity test, Χ^2^ = 17.10, *p* = 0.002, *I* ^*2*^ = 77%). After using the random-effects model to merge the MD data, the pooled MD was −10.96 (95% CI −16.31 to −5.62, *p* < 0.0001). This suggested that the AST levels in the experimental cohort were significantly lower than those in the control cohort (Figure 4B).

The two RCTs (Hu et al., 2012; Wang et al., 2018) that compared GGT levels had enrolled 196 participants, with 98 of them participating in the experimental cohort and 98 participating in the control cohort. Heterozygosity was recorded in the two trials (heterozygosity test, Χ^2^ = 18.84, *p* < 0.0001, *I* ^*2*^ = 95%). After using the random-effects model to merge the MD values, the pooled MD was −9.59 (95% CI −30.75 to 11.56, *p* = 0.37). This suggested that there was no significant difference between the experimental and control cohorts (Figure 4C).

#### 3.5.4 Blood lipid indices

**FIGURE 5:**
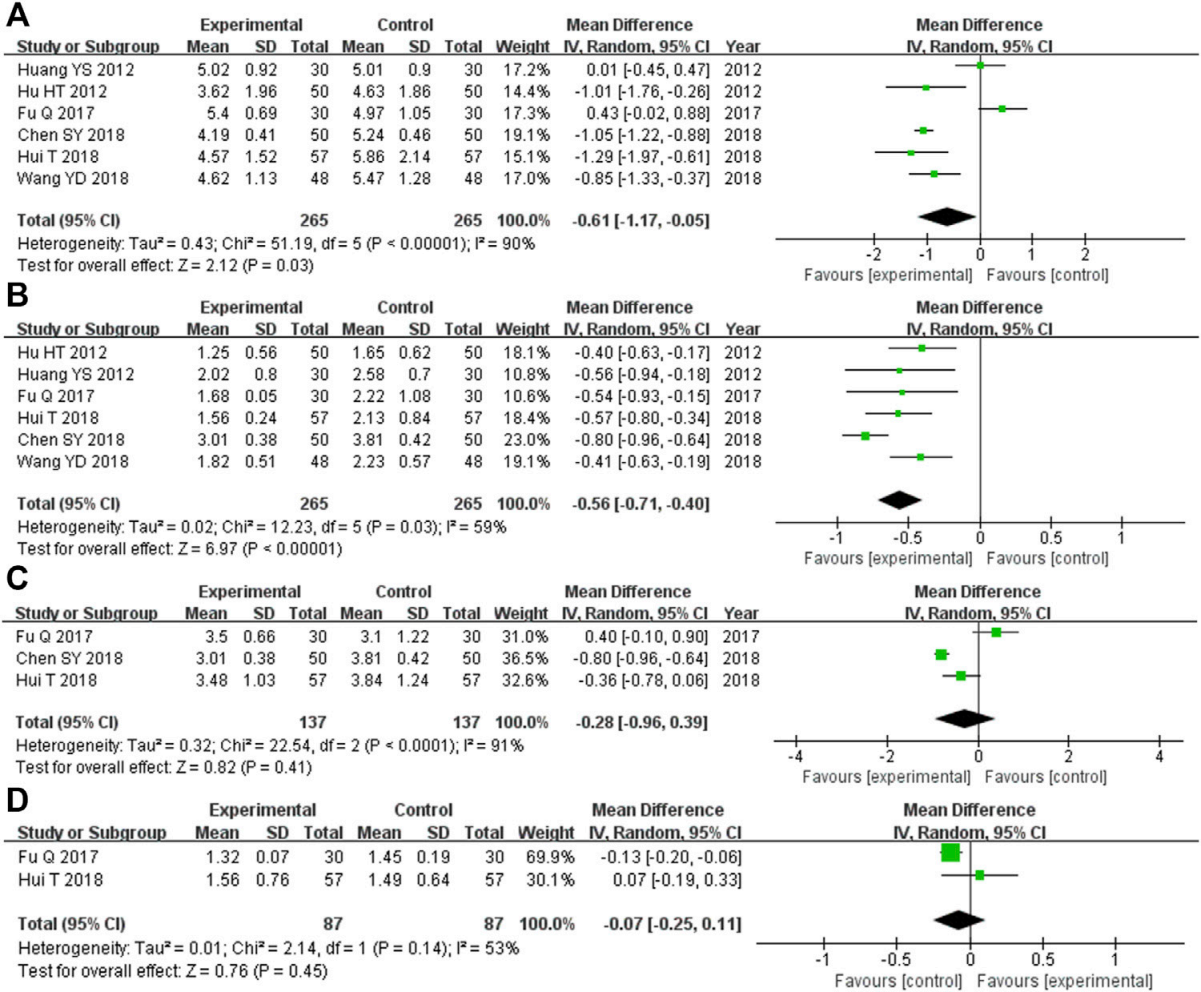
Comparative forest plot: blood lipid indices. **(A)** The pooled MD was −0.61 (95% CI −1.17 to −0.05, *p* = 0.03). TC levels in the experimental cohort were significantly lower than those in the control cohort. **(B)** The pooled MD was −0.56 (95% CI −0.71 to −0.40, *p* < 0.00001). TG levels in the experimental cohort were significantly lower than those in the control cohort. **(C)** The pooled MD was −0.28 (95% CI −0.96 to 0.39, *p* = 0.41). There was no significant difference in the LDL-C level between the experimental and control cohorts. **(D)** The pooled MD was −0.07 (95% CI −0.25 to 0.11, *p* = 0.45). There was no significant difference in HDL-C levels between the experimental and control cohorts. MD, mean difference; CI, confidence interval; TC, total cholesterol; TG, triglyceride; LDL-C, low-density lipoprotein cholesterol; HDL-C, high-density lipoprotein cholesterol.

There were 530 participants enrolled into the six trials (Hu et al., 2012; Huang et al., 2012; Fu, 2017; Chen et al., 2018; Hui, 2018; Wang et al., 2018) comparing TC levels, and among these participants, 265 were in the experimental cohort and 265 were in the control cohort. Heterozygosity was observed in all six of the RCTs (heterozygosity test, Χ^2^ = 51.19, *p* < 0.00001, *I* ^*2*^ = 90%). The pooled MD obtained after using the random-effects model to merge the MD values was −0.61 (95% CI −1.17 to −0.05, *p* = 0.03). This suggests that the TC level was significantly lower in the experimental cohort compared with that of the control cohort (Figure 5A).

The six RCTs (Hu et al., 2012; Huang et al., 2012; Fu, 2017; Chen et al., 2018; Hui, 2018; Wang et al., 2018) that compared the TG levels had enrolled 530 participants, and 265 of these participants were in the experimental cohort, while 265 were in the control cohort. Heterozygosity was observed across all six trials (heterozygosity test, Χ^2^ = 12.23, *p* = 0.03, *I* ^*2*^ = 59%). The pooled MD after applying the random-effects model to merge the MD values was −0.56 (95% CI −0.71 to −0.40, *p* < 0.00001). This demonstrated that TG levels were significantly lower in the experimental cohort compared with those of the control cohort (Figure 5B).

In the three RCTs (Fu, 2017; Chen et al., 2018; Hui, 2018) that compared LDL-C levels, there were 174 participants enrolled, and among them, the experimental and control cohorts had 87 participants each. Heterozygosity was found across the two trials (heterozygosity test, Χ^2^ = 22.54, *p* < 0.0001, *I* ^*2*^ = 91%). The pooled MD was −0.28 (95% CI −0.96 to 0.39, *p* = 0.41) after using the random-effects model to merge the MD values. There was no significant difference between the experimental and control cohorts (Figure 5C).

Overall, 174 participants were enrolled into the two RCTs (Fu, 2017; Hui, 2018) that compared HDL-C levels. Among them, 87 were in the experimental cohort and the other 87 were in the control cohort. Both of these trials showed heterozygosity (heterozygosity test, Χ^2^ = 2.14, *p* = 0.14, *I* ^*2*^ = 53%). The pooled MD was −0.07 (95% CI −0.25 to 0.11, *p* = 0.45) after applying the random-effects model to merge the MD values. This suggested that there was no significant difference between the control and experimental cohorts (Figure 5D).

#### 3.5.5 Blood glucose-related indicators

**FIGURE 6:**
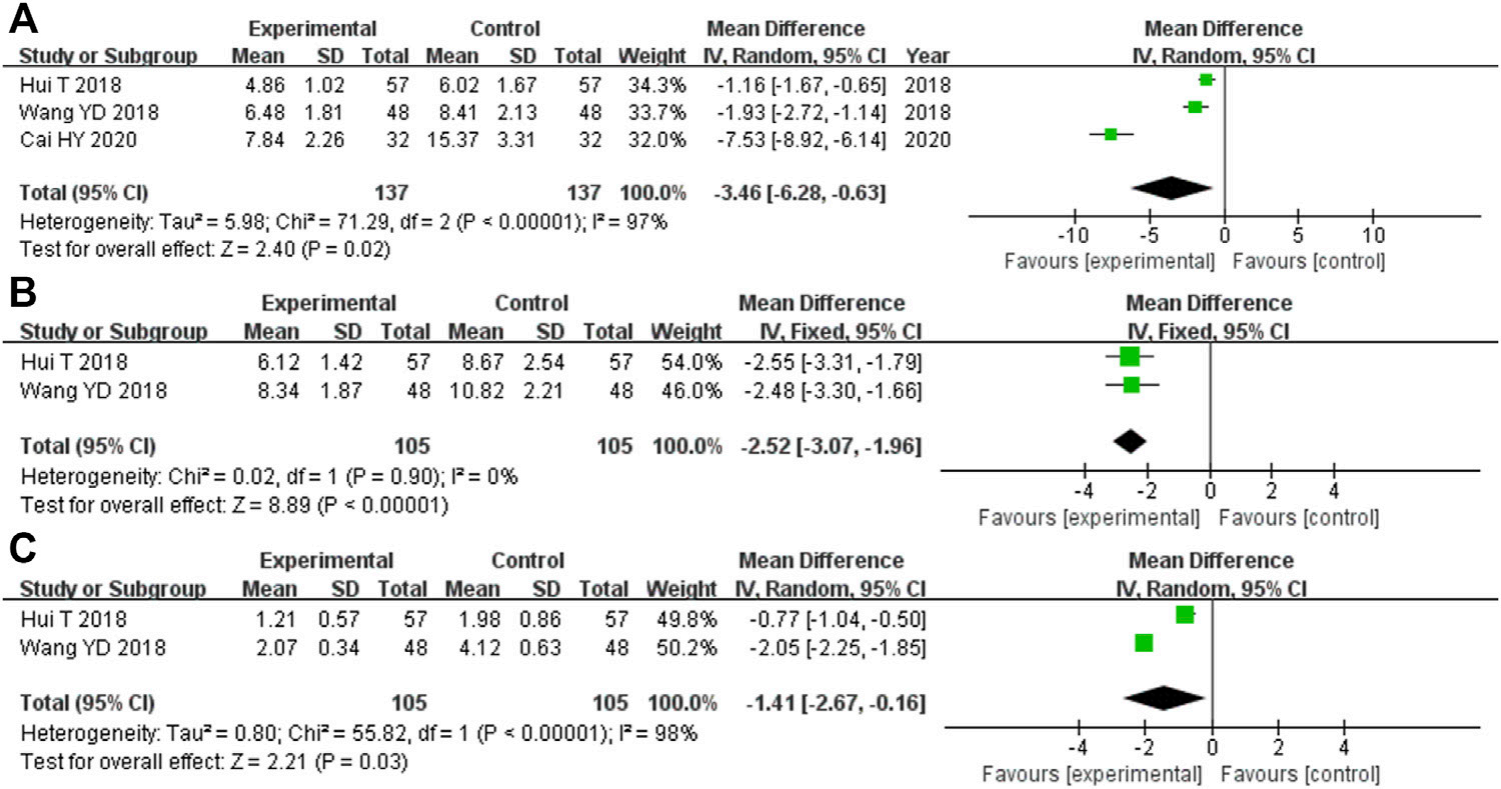
Comparative forest plot: blood glucose-related indicators. **(A)** The pooled MD was −3.46 (95% CI −6.28 to −0.63, *p* = 0.02). FPG was significantly lower in the experimental cohort compared with that in the control cohort. **(B)** The pooled MD was −2.52 (95% CI −3.07 to −1.96, *p* < 0.00001). FINS in the experimental cohort was significantly lower than that in the control cohort. **(C)** The pooled MD was −1.41 (95% CI −2.67 to −0.16, *p* = 0.03). HOMA-IR in the experimental cohort was significantly lower than that in the control cohort. MD, mean difference; CI, confidence interval; FPG, fasting plasma glucose; FINS, fasting serum insulin; HOMA-IR, homeostatic model assessment insulin resistance.

There were 274 participants enrolled into the three trials (Hui, 2018; Wang et al., 2018; Cai, 2020) that compared FPG levels, and 137 of them were randomized to the experimental cohort and 137 were in the control cohort. Heterozygosity was observed in all three RCTs (heterozygosity test, Χ^2^ = 71.29, *p* < 0.00001, *I* ^*2*^ = 97%). The pooled MD after merging the MD values using the random-effects model was −3.46 (95% CI −6.28 to −0.63, *p* = 0.02). This suggested that the FPG levels were significantly lower in the experimental cohort compared with those in the control cohort (Figure 6A).

In the two RCTs (Hui, 2018; Wang et al., 2018) that compared the FINS, there were 210 participants overall, with 105 participants each in the experimental and control cohorts. Both of these trials showed homogeneity (heterozygosity test, Χ^2^ = 0.02, *p* = 0.90, *I* ^*2*^ = 0%). The pooled MD after merging the MD values using a fixed-effects model was −2.52 (95% CI −3.07 to −1.96, *p* < 0.00001). This showed that the FINS was significantly lower in the experimental cohort compared with that in the control cohort (Figure 6B).

The two RCTs (Hui, 2018; Wang et al., 2018) that compared the HOMA-IR had 210 participants enrolled, with 105 in each of the experimental and control cohorts. Heterozygosity was recorded in each of the trials (heterozygosity test, Χ^2^ = 55.82, *p* < 0.00001, *I* ^*2*^ = 98%). After merging the MD values using the random-effects model, the pooled MD was −1.41 (95% CI −2.67 to −0.16, *p* = 0.03). This suggested that the HOMA-IR levels in the experimental cohort were significantly lower than those in the control cohort (Figure 6C).

#### 3.5.6 Adverse reactions

**FIGURE 7:**
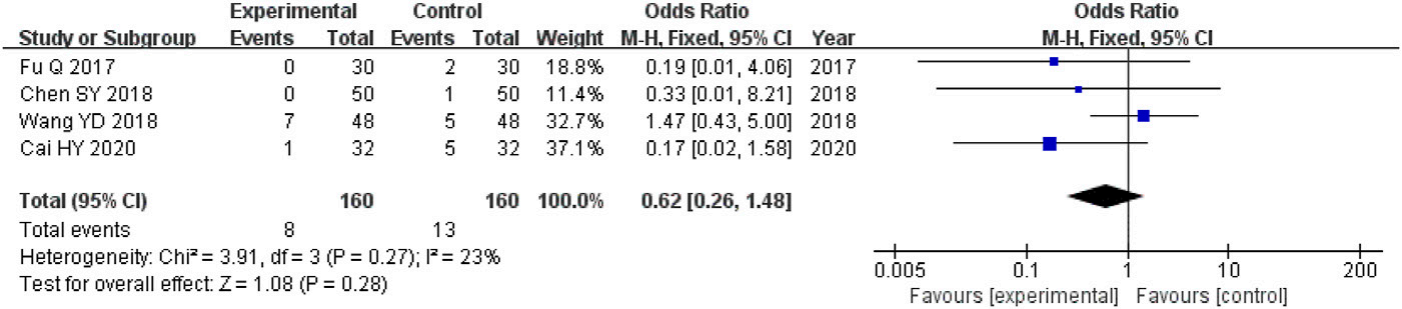
Comparative forest plot: incidence of adverse reactions. The pooled OR was 0.62 (95% CI 0.26–1.48, *p* = 0.28). There were no significant differences in the incidence of adverse events between the experimental and control cohorts.

The four studies (Fu, 2017; Chen et al., 2018; Wang et al., 2018; Cai, 2020) that compared adverse outcomes had enrolled 320 participants, and the experimental and control cohorts had 160 participants each. There was homogeneity across all four of the trials (heterozygosity test, Χ^2^ = 3.91, *p* = 0.27, *I* ^*2*^ = 23%). The pooled OR was 0.62 (95% CI 0.26–1.48, *p* = 0.28) as determined using the random-effects model to merge the OR values. These results showed that there was no significant difference between the experimental and control cohorts (Figure 7).

#### 3.5.7 Publication bias analysis

**FIGURE 8:**
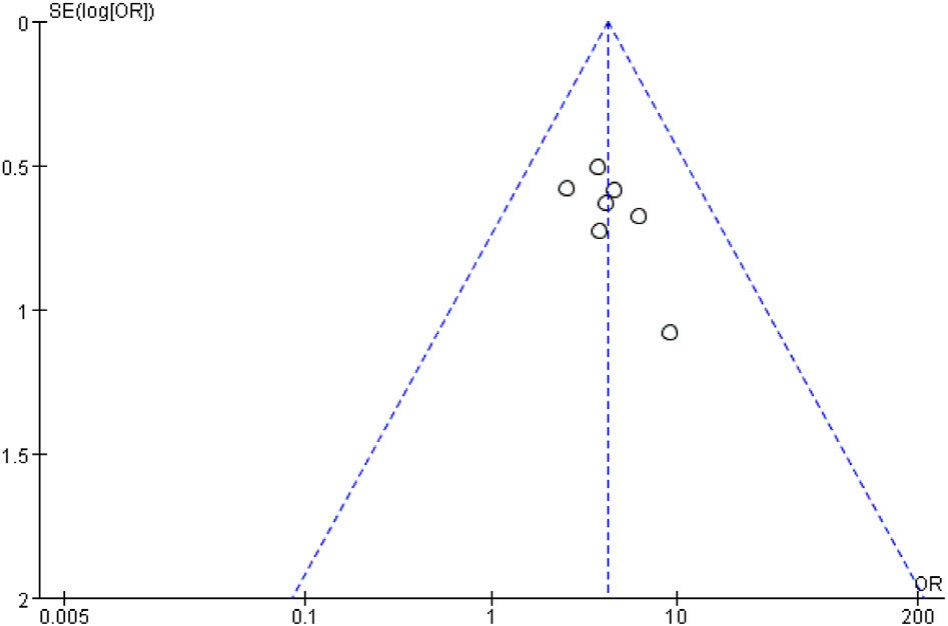
Funnel plot of total clinical effective rate. The funnel plot displayed an inverted and symmetrical funnel shape. Thus, there was no clear evidence of publication bias.

Using a funnel plot, we examined the publication bias of the total clinical effective rate, which was constructed using the OR value of each outcome as the horizontal coordinate and SE [log (OR)] as the longitudinal coordinate. Additionally, the funnel plot showed an inverted but symmetrical form. The findings demonstrated no conclusive proof of publication bias. Figure 8 shows a funnel plot illustrating the total clinical effective rate.

## 4 Discussion

NAFLD is the most prevalent chronic liver disease globally, but there is currently no drug designed specifically to treat this condition. For decades, TCM has been used extensively to treat hepatic disorders in Asia. TCM’s holistic philosophy and differentiation therapy for NAFLD suggest that it has benefits in managing this complicated metabolic condition (Dai X. et al., 2021).

WDD primarily consists of RP, CBT, FAI, PCR, PA, and RG. RP is a JUN (Monarch) herb that has the effect of lowering qi and drying dampness phlegm. CBT is a CHEN (Minister) herb that has the effect of reducing phlegm and opening depression, clearing the stomach, and reducing pressure. Additionally, FAI has the effect of promoting qi, breaking stagnation, reducing phlegm, and resolving masses; PCR has the effect of harmonizing qi, removing phlegm, and dissolving dampness; and PA has the effect of fortifying the spleen and percolating dampness. These latter three herbs are used as ZUO (Assistant) herbs. RG has the effect of replenishing qi and falling the *versus*, which is used as a SHI (Guide) herb (Pu and Hua, 2009; Fu, 2017). The main pathogenesis of NAFLD is weakness in the spleen and stomach, and “phlegm” is the most basic and important pathological factor in NAFLD (Liang et al., 2021). The main pharmacological effects of WDD are regulating qi, reducing phlegm, and benefiting the stomach, which is consistent with the TCM treatment for NAFLD.

However, no systematic review has shown comprehensive evidence for the safety and effectiveness of WDD in treating NAFLD. This systematic review showed a significantly elevated total clinical effective rate in the WDD-treated cohort compared with that in the control cohort (*p* < 0.05). For example, serum ALT and AST levels, TC and TG levels, and HOMA-IR, FINS, and FPG were all significantly decreased in the WDD cohort compared with those in the control cohort (all *p* < 0.05), and there was no significant difference in the incidence of adverse reactions between the two cohorts (*p* > 0.05).

The total clinical effective rate was the most intuitive index in the clinical efficacy evaluation. When comparing the total clinical effective rate between the WDD and the CWMT groups, two studies (Huang et al., 2012; Chen et al., 2018) did not show a considerable difference. When the sample size was increased, this systematic review confirmed that the total clinical effective rate in the WDD cohort was substantially increased compared with that in the CWMT cohort.

Currently, the main laboratory indicators of NAFLD are liver function indices. The meta-analysis results showed that serum ALT and AST levels in the WDD cohort were significantly lower than those in the control cohort, suggesting that WDD has the potential to remarkably improve liver function in NAFLD patients.

NAFLD is a metabolic disorder, which always involves the combination of central obesity, dyslipidemia, and DM (Fujii et al., 2020; Cariou et al., 2021). We also analyzed relevant indicators. Serum TC and TG levels were significantly decreased in the WDD cohort compared with that in the control cohort, which suggests that WDD may substantially improve the blood lipid levels in NAFLD patients. Additionally, we performed a meta-analysis on markers that are associated with blood glucose. FPG, FINS, and HOMA-IR were significantly lower in the WDD cohort compared with those in the control cohort, which demonstrates that WDD may significantly improve the blood glucose-related indicator results in NAFLD patients.

There was no significant variation in the incidence of adverse events between the WDD and the control cohorts. Individual studies demonstrated that severe reactions were relatively mild, and most of them resolved spontaneously, which implies the clinical significance and safety of WDD in treating NAFLD. This systematic review revealed that WDD was a safe and effective treatment for NAFLD, which has advantages over CWMT.

However, this systematic review has some limitations. First, we included studies that used modified WDD, which includes other herbs, in the experimental cohort. Although the other herbs will have some influence on the findings, the function of WDD as the primary component continues to be important. Second, some of the randomization procedures were not explained, and the trials did not include allocation concealment or blinding. Some research methodologies were of poor quality. Although Jadad scores of the included studies were relatively low, we carefully evaluated the literature to ensure that the results are true and credible. More high-quality RCTs are needed to further obtain the best evidence. Finally, the treatment procedures were not standardized, the WDD dose was not constant, and the medicine that was used for the control cohort was not consistent. Because some of the study treatment cycles were rather short, it was not possible to conduct an appropriate risk assessment for the use of WDD in conjunction with NAFLD therapy over the long-term. These biases were evident, which could also affect the robustness of the study findings. However, the primary focus of our research was analyzing the effectiveness of WDD in NAFLD patients. Because there was no specific restriction on the dose, all of the included publications were RCTs, and the diagnosis was uniform. Therefore, the baselines that were considered for inclusion in this research were not substantially different. Additionally, many studies included a chemical analysis of WDD by high-performance liquid chromatography (Shao et al., 2016; Zhang et al., 2021). All of the prescriptions included in this research were formulated by specialists and well-known TCM practitioners in accordance with the Chinese pharmacopeia.

## 5 Conclusion

In summary, WDD is beneficial for treating NAFLD because it was shown to be safe and effective and had a positive impact on the patient’s liver function as well as on their blood glucose and lipid levels. The research is of low quality, the number of participants and the sample size are limited, and additional high-quality RCTs are required that involve multiple centers and have larger sample sizes.
