# Supplementary material for: Wendan decoction in the treatment of nonalcoholic fatty liver disease: A systematic review and meta-analysis
Source: Front Pharmacol. 2022 Oct 17;13:1039611. doi: 10.3389/fphar.2022.1039611 (PMC9618729; doi:10.3389/fphar.2022.1039611)
Supplement: Supplementary file 2 [file DataSheet1.pdf]

Figure S1. Overview of the risk of bias

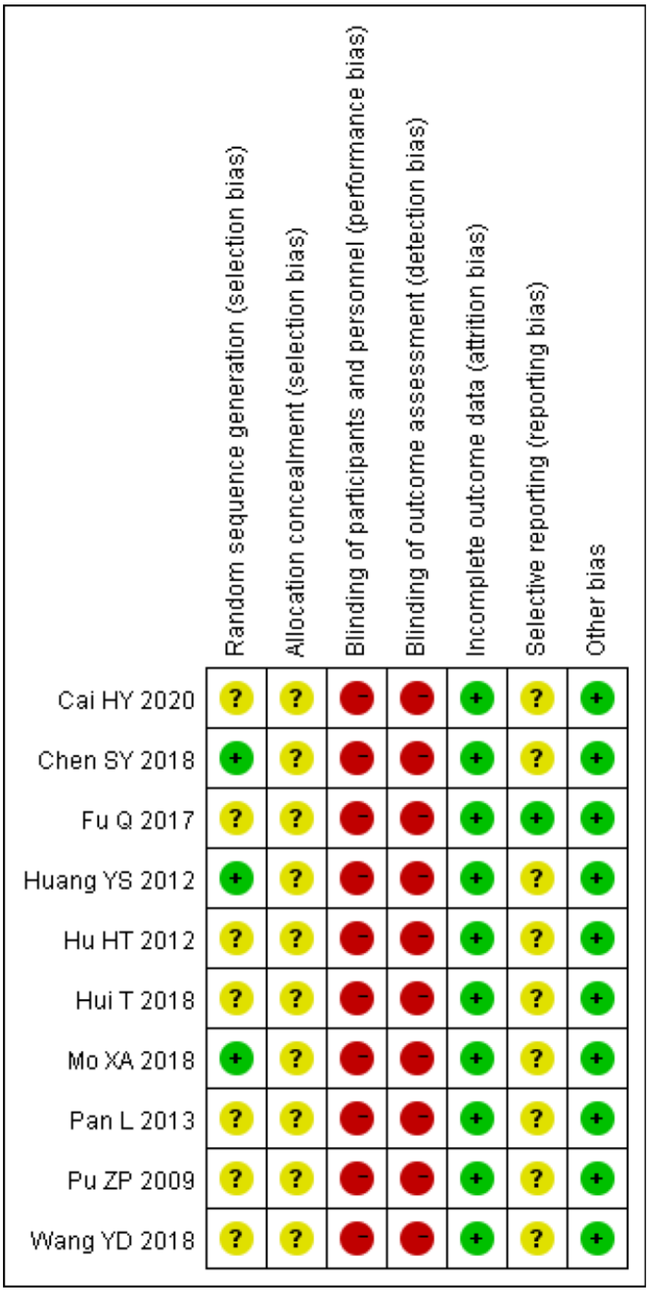

Figure S1. Overview of the risk of bias.

Evaluations of each potential source of bias for each study that was included. Green + denotes a low risk of bias, red - signifies a high risk of bias, and yellow ? indicates an unclear risk of bias.
